# Cellular and temporal expression of NADPH oxidase (NOX) isotypes after brain injury

**DOI:** 10.1186/1742-2094-10-155

**Published:** 2013-12-17

**Authors:** Sean J Cooney, Sara L Bermudez-Sabogal, Kimberly R Byrnes

**Affiliations:** 1 Department of Anatomy, Physiology and Genetics, Uniformed Services University, 4301 Jones Bridge Road, Bethesda, MD 20814, USA

**Keywords:** Animal studies, Models of injury, Oxidative stress, Traumatic brain injury

## Abstract

**Background:**

Brain injury results in an increase in the activity of the reactive oxygen species generating NADPH oxidase (NOX) enzymes. Preliminary studies have shown that NOX2, **NOX**3, and **NOX**4 are the most prominently expressed NOX isotypes in the brain. However, the cellular and temporal expression profile of these isotypes in the injured and non-injured brain is currently unclear.

**Methods:**

Double immunofluorescence for NOX isotypes and brain cell types was performed at acute (24 hours), sub-acute (7 days), and chronic (28 days) time points after controlled cortical impact-induced brain injury or sham-injury in rats.

**Results:**

NOX2, **NOX**3, and **NOX**4 isotypes were found to be expressed in neurons, astrocytes, and microglia, and this expression was dependent on both cellular source and post-injury time. NOX4 was found in all cell types assessed, while NOX3 was positively identified in neurons only, and NOX2 was identified in microglia and neurons. NOX2 was the most responsive to injury, increasing primarily in microglia in response to injury. Quantitation of this isotype showed a significant increase in NOX2 expression at 24 hours, with reduced expression at 7 days and 28 days post-injury, although expression remained above sham levels at later time points. Cellular confirmation using purified primary or cell line culture demonstrated similar patterns in microglia, astrocytes, and neurons. Further, inhibition of NOX, and more specifically NOX2, reduced pro-inflammatory activity in microglia, demonstrating that NOX is not only up-regulated after stimulation, but may also play a significant role in post-injury neuroinflammation.

**Conclusions:**

This study illustrates the expression profiles of NOX isotypes in the brain after injury, and demonstrates that NOX2, and to a lesser extent, NOX4, may be responsible for the majority of oxidative stress observed acutely after traumatic brain injury. These data may provide insight into the design of future therapeutic approaches.

## Background

In the United States alone, there are over 1.7 million cases of traumatic brain injury (TBI) contributing to a third (30.5%) of all injury-related deaths in the United States [1]. Secondary tissue damage and cell death follows the initial mechanical insult, expanding from the primary injury site and contributing to permanent motor, autonomic, and sensory function loss (for a review, see [2]). Inflammation, including activation of microglia, astrogliosis, and oxidative stress, play central roles in this secondary injury [3-6].

Oxidative stress results from an accumulation of reactive oxygen species (ROS), produced by a cascade of reactions that start with the reduction of oxygen to yield superoxide (O_2_^•-^). ROS readily interact with a wide range of other small inorganic and biological molecules, which frequently results in cellular and tissue damage, particularly under acute or chronic inflammatory conditions [7]. A major source of ROS in the injured nervous system is the nicotinamide adenine dinucleotide phosphate (NADPH) oxidase (NOX) enzyme. All NOX family members are transmembrane electron carriers that use cytosolic NADPH as an electron donor and transport electrons through flavin adenine dinucleotide (FAD) and membrane-embedded hemes to reduce oxygen to superoxide. Superoxide largely dismutates to hydrogen peroxide (H_2_O_2_), catalyzed by superoxide dismutase or spontaneously at low pH [7,8]. Furthermore, peroxide and superoxide can react to produce hydroxyl radicals (HO^•^) in the presence of metal ions. Additionally, superoxide reacts with nitric oxide to form the highly reactive intermediate peroxynitrite.

There are seven known isotypes of the catalytic NOX subunit: NOX1, NOX2, NOX3, NOX4, NOX5, DUOX1, and DUOX2. Cellular expression of NOX in the brain, as well as the rest of the body, appears to be isotype dependent. NOX2 is the primary phagocytic oxidase [9-14]; NOX1 and 2 have been shown to be expressed in neurons [10,14,15], smooth muscle cells [9,16], and microglia [9-14]. Savchenko et al. demonstrated that expressions of NOX1 and NOX2 are altered in microglia and neurons after stimulation *in vitro*[10]. Studies have also shown that NOX2 is up-regulated in microglia upon activation in cases of multiple sclerosis [11], ischemia [14], and TBI [17]. Moreover, we have demonstrated that NOX2 components are up-regulated in the spinal cord after injury [18-20]. NOX3 is less studied, but has been shown primarily in the vestibular system to date [21]. NOX4, which does not require any cytosolic components and is constitutively active, is expressed highly in the kidney [22] and vascular smooth muscle cells [16], and to a lesser extent in neurons [23] and astrocytes [24]. Finally, NOX5 is expressed in fetal tissues and spleen [9], while DUOX1 and 2 are highly expressed in the thyroid where they produce H_2_O_2_[7].

Numerous studies have shown evidence of oxidative stress and ROS-mediated damage after TBI, although the source of these ROS is unclear. While some of the acute ROS production may be a result of mitochondrial dysfunction [25], NOX is also likely to play a role. NOX activity has been shown to have an early peak at 1 hour after controlled cortical impact (CCI) injury in mice, followed by a secondary peak between 24 and 96 hours post-injury [26,27]. NOX2 specifically, as well as ROS activity, have been identified in microglia, neurons, and astrocytes, at 48-hours post-CCI injury in mice [17]. In a diffuse brain injury study, NOX2 expression and activity was elevated in the hippocampus between 48 and 72 hours post-injury [28].

While most studies have investigated acute NOX activity, few studies have shown evidence of chronic NOX activity in the brain and spinal cord after injury and oxidative stress [19,29,30]. Further, to date, the temporal and cellular expression of NOX isotypes in the brain and their potential role in oxidative stress after injury have not been clarified. Our preliminary studies have shown that NOX2, **NOX**3, and **NOX**4 are the principle isotypes expressed in the central nervous system. Therefore, the aim of this study was to identify the temporal profile and cellular localization of the NOX2, **NOX**3, and **NOX**4 isotypes in the normal and injured brain. We now show that there is temporal and cellular differentiation in the localization of NOX isotypes in the brain, and that NOX-induced ROS production may stem from different cells at different time points. Further, we show that inhibition of NOX, both non-specifically and specifically for NOX2, can inhibit microglial pro-inflammatory responses and reduce microglial-induced cellular toxicity.

## Methods

### Animal handling and surgical methods

Adult male Sprague Dawley rats (275–325 g) were used for all experiments. Rats were dual housed and received food and water *ad libitum* with a 12:12 hour light cycle. A total of 23 adult male Sprague Dawley rats were used for this study. All experiments complied fully with the principles set forth in the “Guide for the Care and Use of Laboratory Animals” prepared by the Committee on Care and Use of Laboratory Animals of the Institute of Laboratory Resources, National Research Council (DHEW pub. No. (NIH) 85-23, 2985) and were approved by the Uniformed Services University Institutional Animal Care and Use Committee.

Moderate CCI injury was performed using the Leica Impact One (Leica Microsystems, Buffalo Grove, IL, USA), as previously described with modification [31]. Briefly, rats were anesthetized with isoflurane evaporated in a gas mixture containing 70% N_2_O and 30% O_2_ and administered through a nose mask (induction at 4% and maintenance at 2%). Animals were placed into a stereotaxic frame and a 5 mm craniotomy was made using a 5 mm tip diameter microdrill trephine (Fine Science Tools, Foster City, CA, USA) on the central aspect of the left parietal bone, at stereotaxic coordinates Bregma -3 mm posterior, -2.5 mm lateral. Moderate-level injury was induced with a 3 mm flat impactor tip at an impact velocity of 5 m/s, deformation depth of 2 mm and dwell time of 200 ms. Sham injured animals underwent the same experimental procedures, but received a craniotomy only. Naïve animals underwent no surgery or anesthesia. Body temperature was monitored and maintained throughout all surgical procedures using a rectal thermometer and heating pad. Following impact or sham-impact, the craniotomy was left unsealed and the incision was closed with sutures. Animals were allowed to recover on heating pads and received acetaminophen (200 mg/kg) in drinking water for 72 hours post-injury.

### Immunohistochemistry

At 24 hours (n = 4 injured, 2 sham), 7 days (n = 5 injured, 2 sham), and 28 days (n = 4 injured, 2 sham) after surgery or 1 week after delivery (naïve; n = 4), rats were terminally anesthetized (sodium pentobarbital solution [Euthasol, Virbac, Ft. Worth, TX, USA], 0.22 mL/kg, I.P.) and intracardially perfused with 100 mL of 0.9% saline followed by 300 mL of 10% buffered formalin. A 5-mm section of brain centered at the lesion epicenter (or equivalent location in sham/naïve tissue) was dissected, post-fixed in 10% buffered formalin overnight, and cryoprotected in 30% sucrose for 48 hours.

Standard hematoxylin and eosin staining (H&E) was performed on serial 20-μm thick coronal sections and photographed at × 20 using NanoZoomer Digital Pathology system (Hamamatsu Photonics, K.K., Japan).

Standard single or double fluorescent or diaminobenzidine (DAB)-based immunohistochemistry on serial 20-μm thick coronal sections was performed as described previously [18]. Antibodies included NOX2/gp91^PHOX^ (1 μg/mL; Abcam, Cambridge, MA, USA), NOX3 (18 μg/mL, Santa Cruz Biotechnology, Santa Cruz, CA, USA), NOX4 (1 μg/mL, Abcam), Iba-1 (1 μg/mL, Wako, Richmond, VA, USA), NeuN (1:100, Millipore, Billerica, MA, USA), GFAP (1:1,000, Abcam), and CD11b (2 μg/mL, Serotec, Oxford, UK) .

Appropriate secondary antibodies linked to AlexaFluor dyes (Invitrogen, Carlsbad, CA, USA) were incubated with tissue sections for 1 hour at room temperature. Slides were coverslipped using mounting media containing DAPI to counterstain for nuclei (Vector Labs, Burlingame, CA, USA). For NOX3 immunohistochemistry, the Vector ABC kit with DAB and/or NovaRed reaction products were utilized due to poor resolution with secondary fluorescence (Vector Labs).

To ensure accurate and specific staining, negative controls were used in which the primary antibody was not applied to sections from injured tissue, and only staining of cells that were double-labeled with expected cell markers or had expected labeling patterns (i.e., classic microglia morphology) was confirmed as positive labeling. In addition, western blotting for each NOX isotype showed a single band, suggesting specificity of the antibody (data not shown).

Immunofluorescence was detected using an Olympus DP72 microscope with Olympus cellSens microscopy software (Olympus, Center Valley, PA, USA). Scion Image Analysis (http://rsb.info.nih.gov/nih-image) was used to assess pixel density of resultant NOX2^+^ immunolabeling using stain intensity as threshold. Measurement was performed on × 5 images obtained with the NanoZoomer Digital Pathology System in the perilesional region in a 4.23 mm^2^ rectangular region of interest centered on the lesion epicenter. The size of the region of interest was maintained for all quantification of images. For all quantitative and qualitative assessments, a minimum of five images were collected from regular intervals throughout the 5 mm length of the lesion, to be representative of the lesion and peri-lesional region.

Because no marked differences were observed between sham-injured and naïve animals at any time point, data from these animals was combined into a single sham/naïve group for all comparisons and analyses.

### *In vitro* experiments

The BV2 microglial cell line (a gift from Dr. Carol Colton) was cultured and replated to wells at passage 14 to 19. The PC12 neuronal cell line (ATCC, Manassas, VA, USA) was cultured and replated to wells at passage 3 to 5. Primary cortical astrocytes were obtained from neonatal rat pups as previously described [32]. Briefly, the whole brain was dissected from P2 Sprague Dawley rat pups and homogenized. Mixed glial cultures were then incubated for 8 to 10 days at 37°C with 5% CO_2_ in Dulbecco’s modified Eagle media (Invitrogen) with 10% fetal calf serum (Hyclone, Logan, UT, USA), 1% L-glutamine (Invitrogen), 1% sodium pyruvate (Invitrogen), and 1% Pen/Strep (Fisher, Pittsburgh, PA, USA). After the initial incubation, the cells were shaken for 1 hour at 100 rpm and 37°C to remove microglia. The flasks were then returned to the shaker for 3 days (with media changes every morning), at 200 rpm and 37ºC. After shaking, astrocytes were removed from the flasks by trypsinization and transferred to new flasks. Primary oligodendrocytes were cultured similarly, as previously described [33]. Briefly, the whole brain from neonatal rat pups was isolated and used to generate mixed glial cultures. At 10 days after culture, flasks were shaken overnight at 200 rpm. Cells were then plated onto poly-L-lysine coated plates in DMEM as described above, containing 30 ng/mL of T3 (Sigma, St. Louis, MO, USA) to generate mature oligodendrocytes.

### Treatments

BV2 cells and astrocytes were treated with lipopolysaccharide (LPS; 100 ng/mL, Sigma), Phorbol-12-Myristate-13-Acetate (PMA; 10 to 500 ng/mL in DMSO, Santa Cruz Biotechnology) or control vehicle media for 24 hours. NOX inhibitors diphenyleneiodonium (DPI; 100 nM to 10 μM in DMSO, Sigma) or gp91ds-tat peptide (1 to 100 μM, AnaSpec, Fremont, CA, USA) were applied 1 hour prior to LPS or PMA. Control for NOX inhibitors included 1% DMSO in media or scrambled ds-tat (10 μM, AnaSpec). All drugs were prepared and stored according to the manufacturer’s guidelines.

### Co-culture

PC12 media was replaced with conditioned media from BV2 cells that were treated with LPS or control media for 24 hours. After 24 hours in BV2 media, cells were fixed with 4% paraformaldehyde and immunostained as described above.

For oligodendrocyte co-culture, BV2 cells were cultured in 24-well plate Transwell slips for 24 hours with LPS or vehicle. After 24 hours, Transwells were transferred to 24-well plates containing oligodendrocytes and cultured for an additional 24 hours. Cell death of oligodendrocytes was measured by lactate dehydrogenase release assay following removal of Transwells containing microglia (CytoTox96TM cytotoxicity assay, Promega, Madison, WI, USA). Measurements are presented as percentage of maximal lactate dehydrogenase (LDH) release following treatment of oligodendrocytes with lysis solution (following manufacturer’s instructions).

### Immunocytochemistry

BV2, astrocyte and PC12 cells were fixed with 4% paraformaldehyde at 24 hours after treatment and immunostained as described above for NOX isoforms. All immunocytochemical assessments were made from three separate cultures for each antibody and cell type.

### Nitric oxide (NO) assay

Briefly, BV2 microglia were pre-treated for 1 hour with gp91ds-tat (1 to 100 μM) or DPI (100 nM to 10 μM), or respective controls prior to stimulation with LPS (100 ng/mL), and incubated at 37°C and 5% CO_2_ for 24 hours. NO^•^ production was assayed using the Griess Reagent Assay kit (Invitrogen), according to the manufacturer’s instructions [34]. Colorimetric changes in 96-well plates were assessed using a Chroma Plate Reader (Midwest Scientific, St. Louis, MO, USA) at 545 nm. Data are presented as percentage of control-treated values.

### Measurement of intracellular ROS

BV2 microglia were pre-treated for 1 hour with gp91ds-tat (1 to 50 μM) or DPI, or respective controls, and exposed to PMA or LPS stimulation. Cells were then incubated at 37°C and 5% CO_2_ for 24 hours. The cells were incubated with 10 μM 2’ ,7’-dichlorodihydrofluorescein diacetate (Molecular Probes, Eugene, OR, USA) for 45 min at 37°C in 5% CO_2_. Fluorescence was measured using excitation and emission wavelengths of 490 and 535 nm, respectively. Data are presented as percentage of control-treated values.

### Statistics

Quantitative data are presented as mean ± standard deviation. Pixel density was obtained in at least five separate sections by an investigator blinded to treatment group. Quantitative cell culture data were obtained from three separate experiments and presented as an average of those data. Data were analyzed using one-way ANOVA with Tukey post-test. All statistical tests were performed using the GraphPad Prism Program, Version 5.0 for Windows (GraphPad Software, San Diego, CA, USA). A *P* value <0.05 was considered statistically significant.

## Results

**Figure 1:**
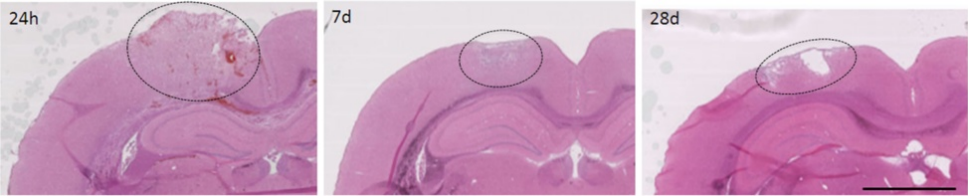
**H&E tissue staining showing a steady lesion evolution over time.** Injury site (dotted circles) can be observed at 24 hours, 7 days, and 28 days post-injury. Images are representative from within the 5 mm segment encompassing the lesion site. Bar = 2.5 mm.

NOX isotypes were evaluated in tissue from sham or naïve rats or from rats that had received a CCI injury 24 hours, 7 days, or 28 days previously. H&E tissue staining showed an evolution of the lesion over time (Figure 1). Tissue showed an initial swelling, followed by slight cavitation and tissue disorganization, then larger cavitation at 28 days post-injury. All measurements of NOX isotypes were made in the lesion epicenter and perilesional region surrounding these sites (Figure 1).

### Microglia/macrophages

**Figure 2:**
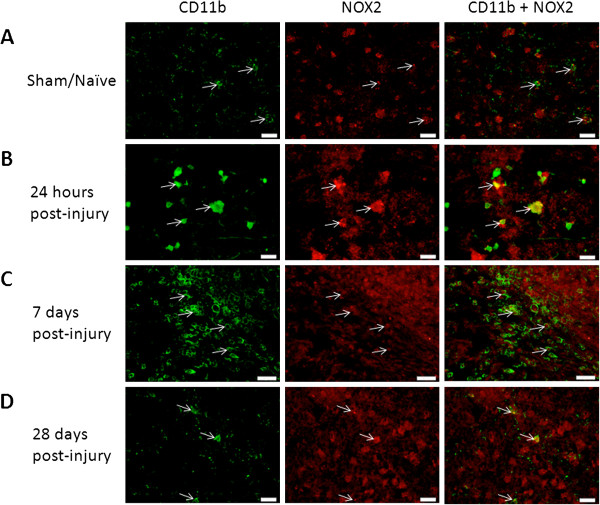
**Microglia were positive for NOX2 ipsilateral to the injury site.** Microglia were labeled with CD11b (green) and immunostained with an antibody against NOX2 (red) in sham-injured tissue and at 24 hours, 7 days, and 28 days post-injury. Co-labeled cells are indicated with arrows. NOX2^+^ cells increased by 24 hours post-injury and increased further by 7 days post-injury, but by 28 days post-injury staining resembled sham. Bar = 20 μm **(A, B, D)**; 50 μm **(C)**.

In uninjured brain, CD11b^+^ microglia were small and few in number; many were NOX2^+^ (Figure 2). By 24 hours post-injury, CD11b^+^ cells showed signs of activation, including increased cell size and number. At this point, most, but not all were also NOX2^+^, resulting in an increase in NOX2^+^ labeling in comparison to sham/naïve tissue. By 7 days post-injury, CD11b^+^ cell number was elevated and NOX2 staining appeared to be similarly increased, although, again, not all CD11b^+^ cells were also NOX2^+^, and many NOX2^+^ cells were not CD11b^+^. By 28 days, the number of CD11b^+^ cells had reduced to 24-hour post-injury levels, and NOX2 staining decreased to resemble 24-hour levels as well.

**Figure 3:**
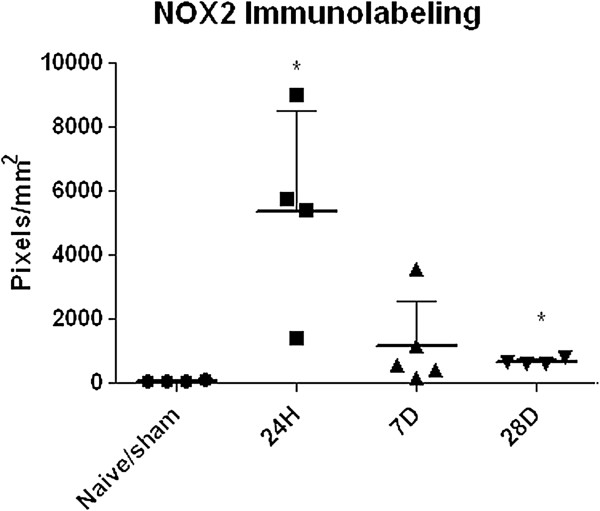
**NOX2 expression increases significantly ipsilateral to the injury site.** Immunofluorescence detection for NOX2 was performed on tissue sections from naïve/sham animals and animals at 24 hours, 7 days, and 28 days after moderate brain injury. Protein expression was measured from the midline to the edge of the injury site by densitometry analysis using stain intensity threshold. NOX2 staining increased by 24 hours post-injury, and was reduced at 7 and 28 days post-injury, although it remained significantly greater than naïve/sham at 28 days. Points represent individual measurements; line at mean and SD. *P <0.05 in comparison to naïve/sham.

Quantitation of NOX2 was performed in tissue from sham/naïve, 24 hour, 7 day, and 28 day injured rats. A significant increase in NOX2 was observed at 24 hours (F_3,9_ = 4.331, *P* <0.05; Figure 3). By 7 days, the amount of NOX2 immunolabeling had reduced. Further reduction was observed at 28 days, but was still significantly elevated over sham. CD11b^+^/NOX3^+^ cells were difficult to identify in brain tissue, so the data was inconclusive (data not shown).

**Figure 4:**
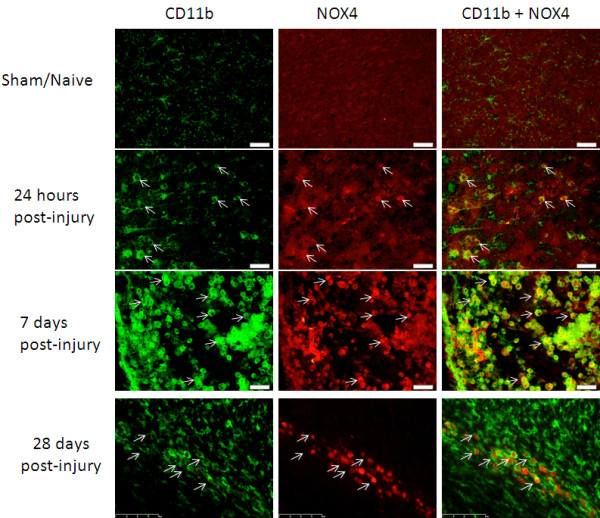
**Microglia were NOX4**^**+ **^**ipsilateral to the injury site.** Cells labeled with the microglial marker CD11b (green) were not NOX4 (red) positive in naïve animals. By 24 hours and 7 days post-injury, some cells positive for CD11b were also positive for NOX4 (arrows). By 7 days post-injury, all CD11b^+^ cells in the injury site were positive for NOX4. At 28 days, not all of the CD11b^+^ cells were NOX4^+^. Bar = 50 μm.

Unlike NOX2, small, sparsely arranged CD11b^+^ cells from sham/naïve tissue were NOX4^-^ (Figure 4). By 24 hours post-injury, the number and size of CD11b^+^ cells was increased, and double staining with NOX4 was frequently observed. NOX4 staining, like NOX2, also increased by 7 days post-injury, and most of the CD11b^+^ cells were now NOX4^+^. By 28 days, both CD11b and NOX4 staining decreased to resemble 24-hour post-injury tissue, and demonstrated several CD11b cells that were not positive for NOX4.

**Figure 5:**
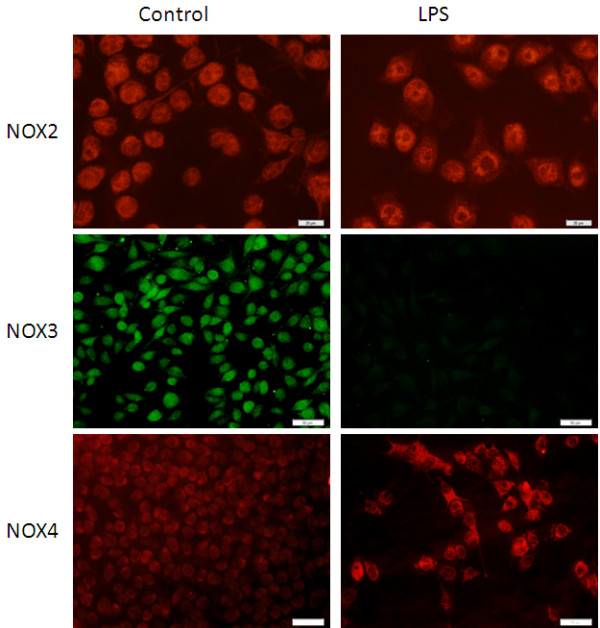
**Microglia show expression of NOX2, NOX3, and NOX4 *in vitro*.** After stimulation with LPS, the distribution of NOX2 changes to surround the nucleus. In contrast, NOX3 staining disappears and the intensity of NOX4 staining increases with LPS stimulation. Microglia stained with NOX2 and NOX4 were stimulated for 24 hours and microglia stained with NOX3 were stimulated for 2 hours. For NOX2, bar = 20 μm. For NOX3 and NOX4, bar = 50 μm.

*In vitro,* unstimulated microglia demonstrated NOX2, NOX3, and NOX4 staining (Figure 5). The intensity of NOX2 and NOX4 staining increased in microglia stimulated with LPS, while NOX3 staining decreased. The distribution of NOX2 changed from the entire cell body in unstimulated microglia to a dense ring surrounding the nucleus in LPS stimulated microglia.

### Neurons

**Figure 6:**
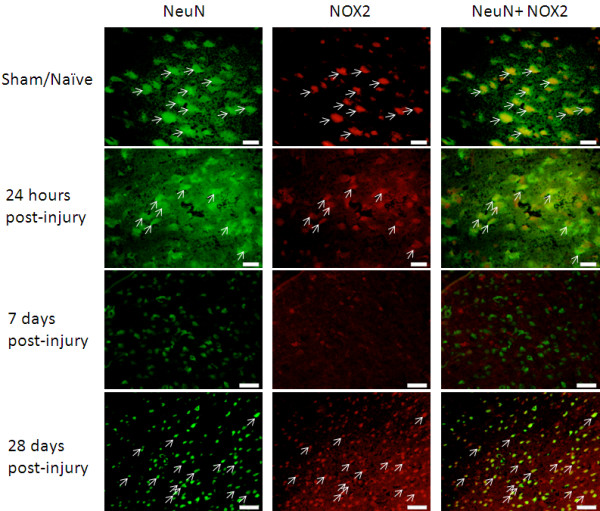
**Neurons expressed NOX2 in the brain ipsilateral to the injury site.** Cells positive for NeuN were also positive for NOX2 in naïve tissue and at 24 hours and 28 days post-injury, but not at 7 days post-injury. Arrows indicate double-labeled cells. Bar = 20 μm (naïve, 24 hours); 50 μm (7 days, 28 days).

In both uninjured and injured brains, the majority of NeuN^+^ cells were NOX2^+^ (Figure 6). The relative amount of staining remained unchanged at 24 hours post-injury, but at 7 days post-injury NeuN^+^/NOX2^+^ were no longer observed, despite sustained presence of NeuN^+^ cells. NOX2 staining returned to NeuN^+^ cells by 28 days post-injury to the level observed in sham/naïve and 24-hour post-injury tissue.

NeuN^+^ cells in uninjured tissue were also NOX3^+^ (data not shown) and remained so through all post-injury time points, with no obvious alterations in expression profile.

**Figure 7:**
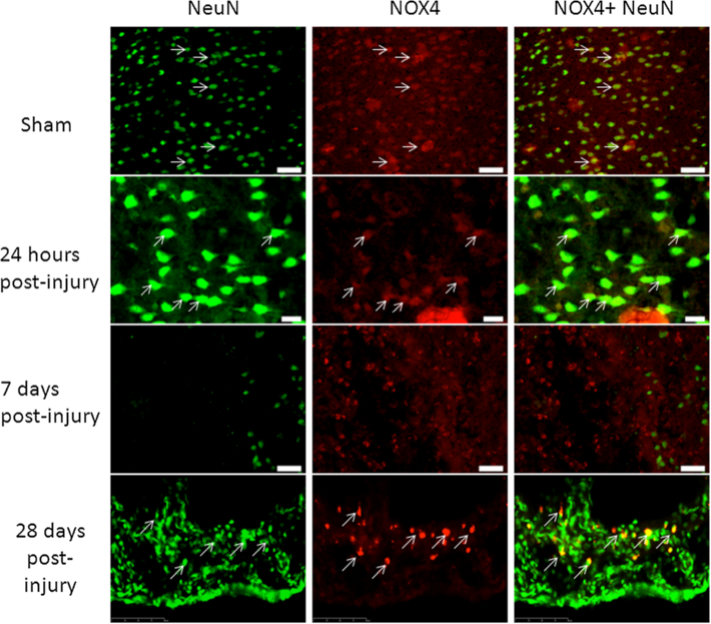
**Neurons were positive for NOX4 in the brain ipsilateral to the injury site.** NeuN^+^ (green) cells were also immunolabeled with NOX4 (red) in sham-injured brains, at 24 hours and at 28 days post-injury, but not in 7 days post-injury brains. Arrows indicate double-labeled cells. Bar = 50 μm (sham and 7 days); 20 μm (24 hours).

Like NOX2, NeuN^+^ cells were found to be NOX4^+^ in uninjured tissue, with most, although not all, NeuN^+^ cells showing NOX4 immunostaining (Figure 7). Brain tissue remained NOX4^+^/NeuN^+^ at 24 hours, with no marked change in the number of cells showing double staining. However, like NOX2, NeuN^+^ cells appeared to lose NOX4 staining at 7 days post-injury. By 28 days post-injury, NOX4 staining returned to NeuN^+^ cells, at levels similar to that seen in sham/naïve tissue.

**Figure 8:**
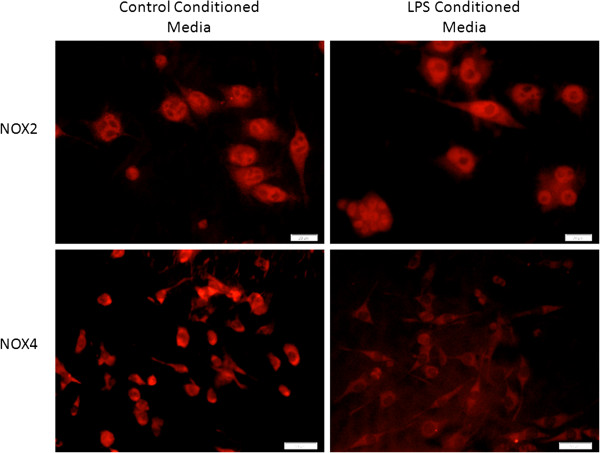
**PC12 neuronal-like cells NOX2, NOX3, and NOX4 *in vitro.*** PC12 cells were cultured in conditioned media from control or LPS-stimulated microglia for 2 hours (NOX2) or 24 hours (NOX4). After addition of LPS-stimulated microglial media, the intensity of NOX2 staining remained the same, but the intensity NOX4 decreased in the neurons. Bar = 20 μm (NOX2); 50 μm (NOX4).

PC12 neuronal like cells were cultured in conditioned control microglial media or in media from microglia that were treated with LPS to test NOX expression in these cell types. NOX2 labeling was consistent in both treatments. However, NOX4 labeling decreased in PC12 cells that were cultured in LPS-treated microglial media (Figure 8).

### Astrocytes

**Figure 9:**
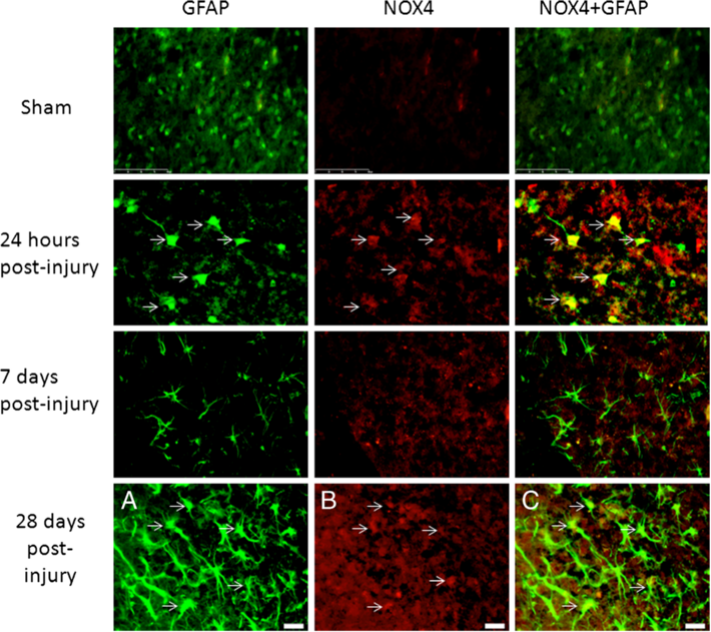
**Astrocytes were positive for NOX4 in the brain ipsilateral to the injury site.** GFAP^+^ (green) cells were not immunolabeled with NOX4 (red) in sham-injured brains or at 7 days post-injury, but were observed at 24 hours and 28 days post-injury (red). Arrows indicate double-labeled cells, which appear as yellow (far right panels). Bar = 50 μm (sham and 7 days); 20 μm (24 hours, 28 days).

Neither injured nor uninjured brain sections showed positive labeling for NOX2 or NOX3 in GFAP^+^ cells in the brain (data not shown). However, at both 24 hours and 28 days post-injury, there was co-labeling of GFAP and NOX4 (Figure 9). Interestingly, no double labeling of NOX4 and GFAP was observed in sham/naïve tissue or at 7 days post-injury.

**Figure 10:**
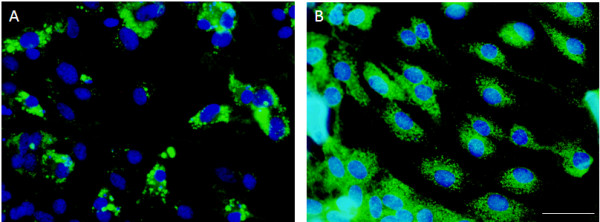
**Astrocytes were positive for NOX2 (A) and NOX4 (B) *in vitro.*** Astrocytes were cultured for 24 hours with or without LPS stimulation. Astrocyte expression of NOX2 and NOX4 did not change with LPS stimulation **(A, B)**. Bar = 50 μm.

*In vitro,* astrocytes were NOX2^+^ and NOX4^+^, but NOX3^-^ (Figure 10). No change in NOX expression was observed over time, nor with LPS treatment.

### *In vitro* experiments

**Figure 11:**
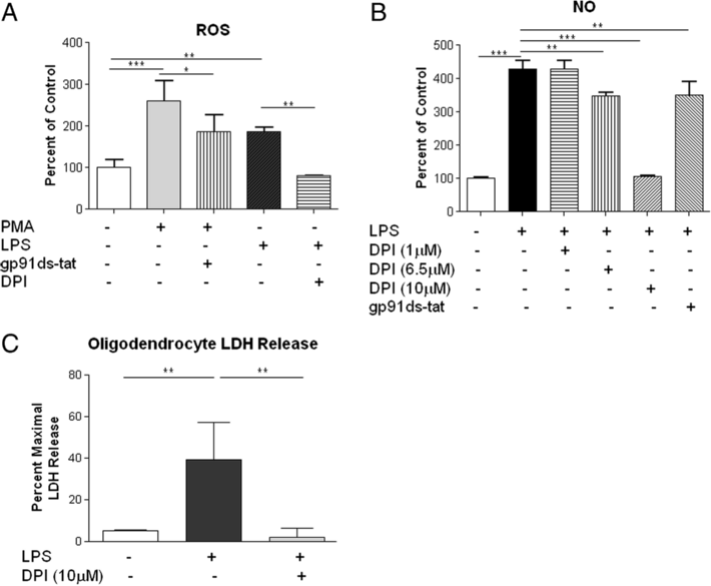
**NOX inhibition reduces microglial activation and toxicity.** BV2 microglial cells incubated with PMA or LPS showed an increase in ROS production **(A)**. Pre-incubation with the NOX2 specific inhibitor gp91ds-tat (10 μM), or the NOX non-specific inhibitor DPI (10 μM), significantly reduced the ROS production induced by PMA and LPS, respectively **(A)**. LPS also induced NO^•^ release from BV2 cells, which was inhibited by gp91-ds-tat (10 μM) and in a dose-dependent fashion by DPI **(B)**. Co-culture of oligodendrocytes with LPS-stimulated BV2 cells for 24 hours resulted in a significant increase in oligodendrocyte cell death, as measured by LDH release **(C)**. Pre-treatment of BV2 cells with DPI (10 μM) for 1 hour prior to LPS administration blocked this toxic effect. Bars represent mean ± SD. *P <0.05; **P <0.01; ***P <0.001.

In order to determine if NOX activity in microglia contributes to the response of microglia following a neuroinflammatory challenge, BV2 microglia cells were cultured in 96-well plates and treated with different NOX inhibitors for 1 hour prior to stimulation with LPS or the protein kinase C activator PMA for a further 24 hours. NO^•^ release and ROS production from microglia were assessed as markers of microglial activation. Microglia showed significant induction of ROS (F_5,16_ = 22.81, *P* <0.001) and NO^•^ (F_5,17_ = 153.1, *P* <0.001) upon LPS and PMA stimulation (Figure 11). Pre-treatment with the non-specific NADPH oxidase inhibitor, DPI, reduced NO^•^ and ROS levels in a dose-dependent manner. Pre-treatment with the NOX2 specific inhibitor peptide, gp91 ds-tat (10 μM), also reduced NO^•^ and ROS induction.

In order to assess the neurotoxic effects of LPS-stimulated microglia in oligodendrocytes, co-cultures were performed with the BV2 microglia cell line. LDH release was measured in oligodendrocytes 24 hours after co-culture with BV2 cells in trans-well plates as a marker of cytotoxicity. Transwell plates containing microglia were removed prior to assessment of cell death. LPS-stimulated microglia induced a significant increase in LDH release from oligodendrocytes 24 hours after co-culture, which was significantly reduced by pre-treatment of microglia with DPI prior to LPS administration (F_2,6_ = 7.2, *P* <0.05).

## Discussion

These data indicate that the expression of the NOX isoforms is dependent on cell type and injury status. We now show that the NOX2 istoype is primarily expressed by microglia and neurons, NOX3 is primarily expressed by neurons, and NOX4 is expressed by all three cell types. Further, NOX2 is the most responsive to injury. Microglia showed the most altered expression of NOX isoforms after injury, which is not unexpected, with the most increase in expression observed at 7 days post-injury, a reported peak in microglial responses after injury [3,35]. Interestingly, quantitation of NOX2 protein showed the greatest increase at 24 hours post-injury, with less elevation by 7 days and 28 days. Because cell type was not taken into account with the quantitation, this suggests that neurons, which showed greater NOX2 immunostaining at 24 hours and none at 7 days, may be the source of much of the NOX2 acutely after injury, and thus much of the ROS production observed at acute time points.

NOX expression has previously been reported to increase in several CNS injury models, including brain [17,27] and spinal cord injury [18,20,36]. In the spinal cord, NOX expression is up-regulated through 6 months after injury in rodents [19], and ROS production has been reported from 6 hours to several months post-injury in humans [35,36]. In the injured brain, NOX activity has been shown to have 2 peaks: the first at approximately 1 hour after injury and the second 24 to 96 hours after injury [17,26-28]. Our results support these studies and suggest that the acute increases may occur in neurons, while the more delayed peaks are likely occurring in microglia/macrophages.

NOX2 was found most prominently in CD11b^+^ macrophages and microglia, and the degree of double staining increased after injury, in agreement with previous studies [17-19]. NOX4 expression was also up-regulated after injury in these cells. This finding was repeated in BV2 cell cultures, an immortalized cell line of microglial cells, which showed similar patterns of NOX2 and 4 expression after LPS stimulation. As we did not differentiate between overall expression and expression per cell, it is possible that the observed increase in NOX2/4 is not necessarily an increase in expression, but an increase in cell number. Future studies will determine if the amount of NOX2/4 per cell is increased or if the overall cell number is the sole reason for this increase.

NOX activity in microglia/macrophages can exacerbate the post-injury inflammatory response via activity on transcription factors that ultimately increase pro-inflammatory cytokine expression [37,38]. Inhibition of NOX activation in microglia, via administration of the non-specific inhibitor apocynin or knockout of the p47^PHOX^ subunit, not only reduces inflammatory responses, but also polarizes microglia into the M2 or alternative activation phenotype [39], which may have significant therapeutic implications. NOX inhibition has also been shown to reduce chemotaxis of neutrophils [40]. Our data support these findings and indicate that inhibition of NOX activity, either generally via the non-specific inhibitor DPI, or specifically of NOX2 using gp91-ds-tat, can reduce aspects known to contribute to inflammation, including NO^•^ and ROS production.

NOX activity is also intimately associated with cellular toxicity (for a review, see [41]). Activated macrophages/microglia release an array of inflammatory cytokines, which induce the release of glutamate, enhancing stimulation on N-methyl-D-aspartate (NMDA) receptors [42]. In addition to the release of free radicals, this cytokine release may result in neuronal and oligodendrocyte toxicity and death. *In vitro* inhibition of NOX activity reduces microglial induction of neuronal [18] and oligodendrocyte cell death [43], which is supported by our study. Co-culture of microglia with oligodendrocytes, which are sensitive to ROS and ROS metabolites produced by microglia [44], demonstrated that non-specific inhibition of NOX resulted in significantly reduced oligodendrocyte cell death. This suggests that NOX activity contributes to the toxicity of M1-activated microglia during a neuroinflammatory challenge and its inhibition may have beneficial consequences after TBI. Inhibition of NOX activity, through non-specific approaches using apocynin or specific inhibitors of NOX2, has been shown to reduce neuronal death after spinal cord injury [45], TBI [26,27], and cortical ischemia [46,47]. In addition, knockout of NOX2 reduces neuronal death after TBI [17]. New research is currently investigating the effects of specific NOX isotype inhibition, with pharmacological or small molecule inhibitors that target NOX2 versus other isotypes [12,48,49]. Qualifying the temporal and cellular localization of NOX2 and other isotypes is an important step in furthering studies that target NOX isotype inhibition.

In addition to microglia and macrophages, neurons and astrocytes were found to express NOX isotypes. In fact, neurons were the only cell type to express all NOX isotypes investigated. Interestingly, NOX2 and NOX4 were only expressed by neurons in sham tissue or acutely after injury, while NOX3 was expressed in neurons constitutively in brain regardless of injury state. Previous studies have shown that after a glutamate injection into the cortex, NOX2 was only observed in neurons at 6 hours and had begun to diminish by 24 hours [50]. NOX2 and NOX4 were also expressed by neurons *in vitro*. On the other hand, astrocytic NOX expression *in vivo* was restricted to the NOX4 isotype. While NOX2 was observed in astrocytes *in vitro*, it is unclear why NOX2 was not observed in these cells *in vivo,* as previous studies have shown that brain astrocytes *in vivo* express NOX2 [51] and may reflect a technical difficulty.

Currently, the function of NOX isotypes in these cell types is not fully understood. For example, neuronal NOX2 has been shown to contribute to both intra- and extracellular release of ROS following NMDA stimulation [10,52] and NOX4 has been observed in spinal cord neurons to play a significant role in post-injury neuropathic pain [53], but the role of NOX3 is unclear. Functionally, NOX2 has been shown to play a role in potentiating inflammation and enhancing the release of matrix metalloproteinases by brain astrocytes [54], while the function of NOX4 in these cells is unclear [55]. Future work will investigate the functional importance of NOX isoform neuronal and astrocyte expression.

## Conclusions

In summary, we now show that NOX isotypes have a temporal and cellular dependence that has not been previously observed, which may suggest different mechanisms of activation of the isotypes in each cell. These data provide support for the observations of ROS-mediated cellular damage and oxidative stress after injury, and may provide insight into the design of future therapeutic approaches for oxidative damage to the injured brain.

## Abbreviations

CCI: Controlled cortical impact; DAB: Diaminobenzidine; FAD: Flavin adenine dinucleotide; H&E: Hematoxylin and eosin staining; LDH: Lactate dehydrogenase; LPS: Lipopolysaccharide; NAPDH: Nicotinamide adenine dinucleotide phosphate; NMDA: N-methyl-D-aspartate; NOX: NADPH oxidase; ROS: Reactive oxygen species; TBI: Traumatic brain injury.

## Competing interests

The authors declare that they have no competing interests.

## Authors’ contributions

SJC carried out all animal studies, tissue preparation and immunostaining, and drafted the manuscript. SLBS carried out all *in vitro* analyses and participated in figure generation and revision of the manuscript. KRB conceived of the study, and participated in its design and coordination, and helped to draft the manuscript. All authors read and approved the final manuscript.
